# QM/MM MD and Free Energy Simulations of G9a-Like Protein (GLP) and Its Mutants: Understanding the Factors that Determine the Product Specificity

**DOI:** 10.1371/journal.pone.0037674

**Published:** 2012-05-18

**Authors:** Yuzhuo Chu, Jianzhuang Yao, Hong Guo

**Affiliations:** 1 Department of Biochemistry and Cellular and Molecular Biology, University of Tennessee, Knoxville, Tennessee, United States of America; 2 National Institute for Mathematical and Biological Synthesis, University of Tennessee, Knoxville, Tennessee, United States of America; 3 University of Tennessee/Oak Ridge National Laboratory Center for Molecular Biophysics, Oak Ridge National Laboratory, Oak Ridge, Tennessee, United States of America; University of Leeds, United Kingdom

## Abstract

Certain lysine residues on histone tails could be methylated by protein lysine methyltransferases (PKMTs) using S-adenosyl-L-methionine (AdoMet) as the methyl donor. Since the methylation states of the target lysines play a fundamental role in the regulation of chromatin structure and gene expression, it is important to study the property of PKMTs that allows a specific number of methyl groups (one, two or three) to be added (termed as product specificity). It has been shown that the product specificity of PKMTs may be controlled in part by the existence of specific residues at the active site. One of the best examples is a Phe/Tyr switch found in many PKMTs. Here quantum mechanical/molecular mechanical (QM/MM) molecular dynamics (MD) and free energy simulations are performed on wild type G9a-like protein (GLP) and its F1209Y and Y1124F mutants for understanding the energetic origin of the product specificity and the reasons for the change of product specificity as a result of single-residue mutations at the Phe/Tyr switch as well as other positions. The free energy barriers of the methyl transfer processes calculated from our simulations are consistent with experimental data, supporting the suggestion that the relative free energy barriers may determine, at least in part, the product specificity of PKMTs. The changes of the free energy barriers as a result of the mutations are also discussed based on the structural information obtained from the simulations. The results suggest that the space and active-site interactions around the ε-amino group of the target lysine available for methyl addition appear to among the key structural factors in controlling the product specificity and activity of PKMTs.

## Introduction

The tails of histone proteins are subject to a variety of post-translational modifications, and these modifications are believed to be a part of the histone code for chromatin regulation [1]. One of such modifications is the methylation of a range of lysine (K) residues on histones, including K4, K9, K27, K36, K79 on histone H3 and K20 on histone H4 [2]. Catalyzed by protein lysine methyltransferases (PKMTs), histone lysine methylations have been found to affect a variety of important biological processes, including heterochromatin formation, X-chromosome inactivation, transcriptional silencing and activation [3], [4]. PKMTs may be classified based on their ability to transfer one, two or three methyl groups from S-adenosyl-L-methionine (AdoMet, the methyl donor) to the ε-amino group of target lysine, a special property of the enzymes that is termed as product specificity [1], [4]. Since different methylation states may lead to different downstream events [5]–[7], it is of fundamental importance to understand the determinant of the product specificity, including the energetic and structural factors that control the product specificity.

A number of experimental [8]–[17] and computational studies [18]–[26] have been performed to understand the origin of the product specificity for PKMTs. Structural and mutational studies have identified a Phe/Tyr switch located at the active site of many SET domain PKMTs [9], [10], [12], [14]. It has been shown previously that the product specificity of the enzyme may depend on whether this position is occupied by a Phe or Tyr residue. Indeed, it has been observed that this position tends to be occupied by a tyrosine residue for mono-methyltransferases (e.g. Y305 in SET7/9 [17] and Y334 in SET8 [10], [13]) and by a phenylalanine for di- or tri-methyltransferases (e.g. F281 in DIM-5 [9]). Moreover, the substitution at the Phe/Tyr switch position could lead to the change of the product specificity that is consistent with the observations on the wild-type enzymes (see above). For example, the F281→Y mutation for DIM-5 altered the enzyme from a tri-methyltransferase to a mono-/di-methyltransferase [9], while the Y305→F mutation for SET7/9 [17] or Y334→F mutation for SET8 [10], [13] was found to switch the enzyme from a mono-methyltransferase to a di-methyltransferase. For H3K9 G9a methyltransferase, the F→Y mutant at the Phe/Tyr switch site (F1152 in G9a) was found to alter the enzyme from a di-methyltransferase to a mono-methyltransferase [14]; the equivalent residue in its closely relative G9a-like-protein (GLP) is F1209. Previous studies on SET7/9, SET8 and DIM-5 [13], [17]–[19] have suggested that this Phe/Tyr switch may affect product specificity through altering the affinity of an active site water molecule, as the dissociation of this water molecule is likely to make it easier for further methyl addition [13], [17]–[19]. It would be of interest to examine whether this is the case for GLP or G9a as well.

**Figure 1:**
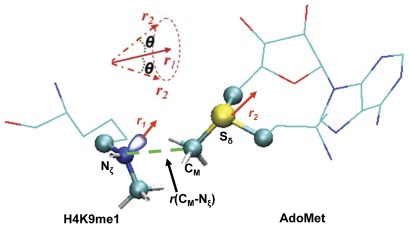
The definition of the structural parameters for monitoring the relative orientation of AdoMet and H3K9me1 [H3K9 and H3K9(me)_2_] in the reactant complex. The efficiency of the methyl transfer may be related to the distributions of *r*(C_M_…N_ζ_) and *θ* in the reactant complexes. *θ* is defined as the angle between the two vectors *r*_1_ and *r*_2_. Here *r*_1_ is the direction of the lone pair of electrons on N_ζ_ and *r*_2_ is the vector pointing from C_M_ to S_δ_. The reaction coordinate for calculating the free energy profiles for the methyl transfers is *R* = *r*(C_M_…S_δ_)−*r*(C_M_…N_ζ_).

**Figure 2:**
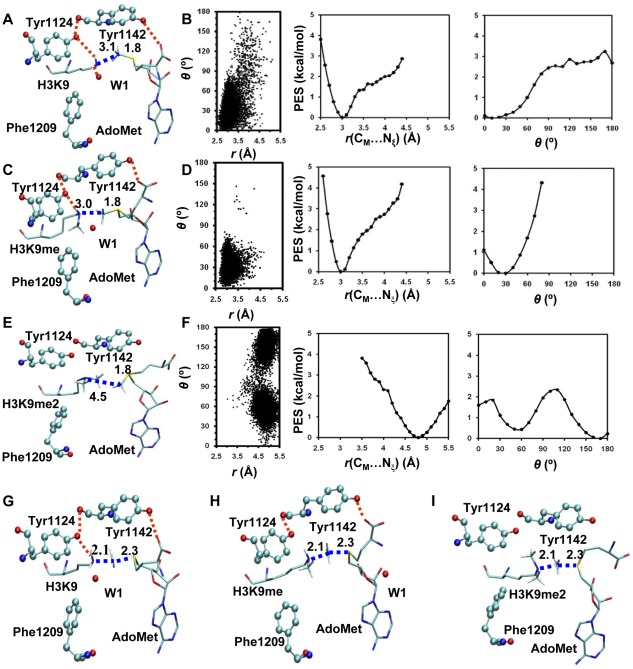
MD results for the WT enzyme (GLP). (A) A representative active-site structure along with the average values of some structural parameters of the reactant complex for the first methyl transfer. GLP is shown in balls and sticks, and AdoMet and the H3K9 sidechain are in sticks. Hydrogen atoms are not shown for clarity except for those on N_ζ_ and transferable methyl group. Hydrogen bonds are indicated by red dotted lines, and the distances related to the reactant coordinate are also shown. (B) Left: the two-dimensional plot of *r*(C_M_…N_ζ_) and *θ* distributions based on the 1.5-*ns* simulations of the reactant complex for the first methyl transfer; Middle: the free-energy change as a function of *r*(C_M_…N_ξ_) obtained from the distributions; Right: the free-energy change as a function of *θ* obtained from the distributions. (C) The active-site structure along with the average values of some structural parameters of the reactant complex for the second methyl transfer. (D) Left: the two-dimensional plot of *r*(C_M_…N_ζ_) and *θ* distributions of the reactant complex for the second methyl transfer; Middle: the free-energy change as a function of *r*(C_M_…N_ξ_) obtained from the distributions; Right: the free-energy change as a function of *θ* obtained from the distributions. (E) The structure of the reactant complex for the third methyl transfer. (F) Left: the two-dimensional plot of *r*(C_M_…N_ζ_) and *θ* distributions of the reactant complex for the third methyl transfer; Middle: the free-energy change as a function of *r*(C_M_…N_ξ_) obtained from the distributions; Right: the free-energy change as a function of *θ* obtained from the distributions. (G) A representative active-site structure along with the average values of some structural parameters near the transition state for the first methyl transfer obtained from the free energy (potential of mean force) simulations. (H) The structure along with the average values of some structural parameters near the transition state for the second methyl transfer. (I) The structure near the transition state for the third methyl transfer. All images were made by VMD [38]. The distances shown on the structures are the calculated average distances from the trajectories over the 50-ps production runs in the corresponding window.

**Figure 3:**
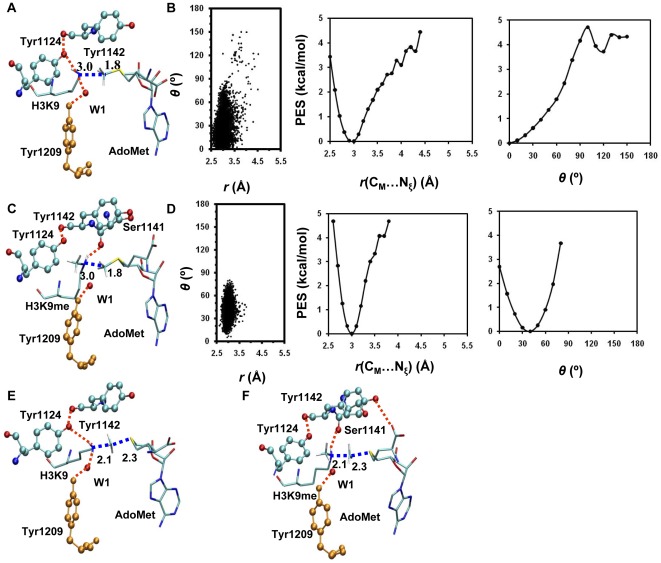
MD results for the F1209Y mutant. (A) A representative active-site structure along with the average values of some structural parameters of the reactant complex for the first methyl transfer. (B) Left: the two-dimensional plot of *r*(C_M_…N_ζ_) and *θ* distributions of the reactant complex for the first methyl transfer; Middle: the free-energy change as a function of *r*(C_M_…N_ξ_) obtained from the distributions; Right: the free-energy change as a function of *θ* obtained from the distributions. (C) The structure of the reactant complex for the second methyl transfer. (D) Left: the two-dimensional plot of *r*(C_M_…N_ζ_) and *θ* distributions of the reactant complex for the second methyl transfer; Middle: the free-energy change as a function of *r*(C_M_…N_ξ_) obtained from the distributions; Right: the free-energy change as a function of *θ* obtained from the distributions. (E) The structure near the transition state for the first methyl transfer. (F) The structure near the transition state for the second methyl transfer.

**Figure 4:**
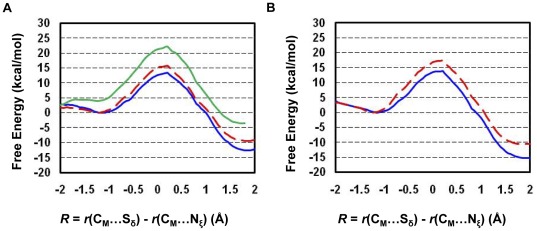
Free energy profiles of methyl transfer processes in WT GLP and the F1209Y mutant. (A) The free energy (potential of mean force) changes for the first, second and third methyl transfers from AdoMet to H3-K9, H3-K9me1 and H3-K9me2, respectively, as a function of the reaction coordinate [*R* = *r*(C_M_…S_δ_)−*r*(C_M_…N_ζ_)] in the wild-type GLP. The first methyl transfer: blue and solid line with a free energy barrier of 13.4 kcal/mol; the second methyl transfer: red and dashed line with a free energy barrier of 15.8 kcal/mol (or about 2.4 kcal/mol higher than that of the first methyl transfer); the third methyl transfer: green and solid line with a free energy barrier of 22.1 kcal/mol (or about 8.7 kcal/mol higher than that of the first methyl transfer). (B) The free energy changes for the first and second methyl transfers as a function of the reaction coordinate in the F1209Y mutant. The first methyl transfer: blue and solid line with a free energy barrier of 13.8 kcal/mol; the second methyl transfer: red and dashed line with a free energy barrier of 17.4 kcal/mol (or about 3.6 kcal/mol higher than that of the first methyl transfer).

**Figure 5:**
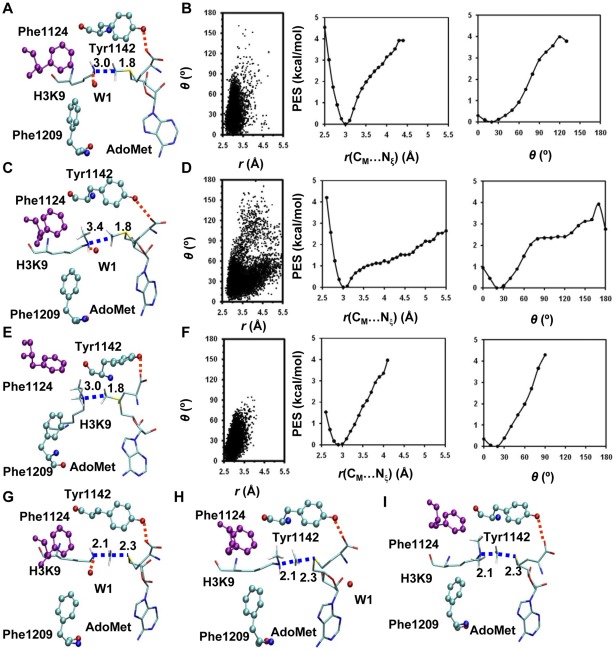
MD results for the Y1124F mutant. (A) A representative active-site structure along with the average values of some structural parameters of the reactant complex for the first methyl transfer. (B) Left: the two-dimensional plot of *r*(C_M_…N_ζ_) and *θ* distributions of the reactant complex for the first methyl transfer; Middle: the free-energy change as a function of *r*(C_M_…N_ξ_) obtained from the distributions; Right: the free-energy change as a function of *θ* obtained from the distributions. (C) The structure of the reactant complex for the second methyl transfer. (D) Left: the two-dimensional plot of *r*(C_M_…N_ζ_) and *θ* distributions of the reactant complex for the second methyl transfer; Middle: the free-energy change as a function of *r*(C_M_…N_ξ_) obtained from the distributions; Right: the free-energy change as a function of *θ* obtained from the distributions. (E) The structure of the reactant complex for the third methyl transfer. (F) Left: the two-dimensional plot of *r*(C_M_…N_ζ_) and *θ* distributions of the reactant complex for the third methyl transfer; Middle: the free-energy change as a function of *r*(C_M_…N_ξ_) obtained from the distributions; Right: the free-energy change as a function of *θ* obtained from the distributions. (G) A representative active-site structure along with the average values of some structural parameters near the transition state for the first methyl transfer. (H) The structure near the transition state for the second methyl transfer. (I) The structure near the transition state for the third methyl transfer.

**Figure 6:**
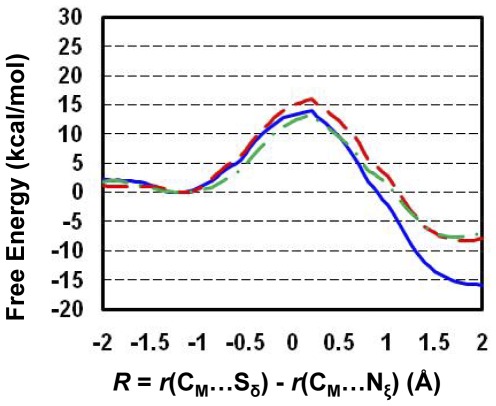
Free energy profiles of methyl transfer processes in the Y1124F mutant. The first methyl transfer: blue and solid line with a free energy barrier of 13.9 kcal/mol; the second methyl transfer: red and dashed line with a free energy barrier of 15.9 kcal/mol; the third methyl transfer: green and dashed line with a free energy barrier of 13.3 kcal/mol.

The alternations of the product specificity of PKMTs by single mutations have also been observed for residues located at some other positions of the active site [9], [15], [17], although detailed studies for the origin of the change of the product specificity due to these mutations are still lacking. For GLP there is a Tyr residue (Y1124) hydrogen-bonding to the methyl accepting nitrogen in the X-ray structures of GLP complexed with the substrate, H3K9me and H3K9me2 peptides [15]; the equivalent residue in G9a is Y1067. It has been found that the Y1067→F mutant for G9a is able to increase the enzyme's ability to add a third methyl group to the target lysine (even though it is not at the Phe/Tyr switch position) and change the enzyme to a tri-methyltransferase [15]. Further studies are still necessary to determine the origin of the product specificity change for this case.

Hybrid quantum mechanical/molecular mechanical (QM/MM) free energy simulations have been performed previously on various SET domain PKMTs, including DIM-5, SET7/9, SET8 and their mutants involving the residue at the Phe/Tyr switch site [18]–[20]. One of the main purposes was to study the energetic origin of the product specificity and identify the structural factors which might control the methylation states of target lysine residues [18]–[20]. For instance, it has been proposed that the existence of a relatively high free energy barrier for one or more methyl transfer processes could stop the continuation of the methyl addition and may therefore determine how the epigenetic marks of lysine methylation are written [18]–[20]. However, questions remain whether the previous conclusions could be extended to mutations involving the residues other than those at the Phe/Tyr switch site. In this paper, the results of the QM/MM molecular dynamics (MD) and free energy simulations are reported for different methyl transfer processes of wild type (WT) GLP as well as its F1209Y and Y1124F mutants. The free energy barriers of the methyl transfer processes obtained from simulations are well correlated with experimental observations of product specificities [14], [15], supporting previous conclusions that the relative free energy barrier for the methyl transfer process may determine, at least in part, the number of methyl groups added by the enzyme [18]–[20]. The dynamic information of reactant complexes gathered from the QM/MM MD simulations were also examined, and the results suggest that the ability of the reactant complex to form the reactive configuration may be used as an important indicator for the product specificity of PKMTs.

## Methods

The QM/MM MD and free energy (potential of mean force) simulations were applied to determine the free energy profiles of different methyl transfer processes catalyzed by WT GLP, F1209Y and Y1124F mutants. MD simulations were also performed on the reactant complexes of the methyl transfers for each system to study their dynamic properties related to the methyl transfers. AdoMet and the target lysine side chains were treated by QM and the rest of the system were treated by MM. The link-atom approach [27] was implemented with CHARMM [28] to separate the QM and MM regions. A modified TIP3 water model [29], [30] was used for the solvent. The stochastic boundary molecular dynamics method [31] was used for the QM/MM MD and free energy simulations. The system was separated into a reaction zone and a reservoir zone, and the reaction zone was further divided into a reaction region and a buffer region. The reaction region was a sphere with radius *r* of 20 Å, and the buffer region had *r* equal to 20 Å≤*r*≤22 Å. The reference center for partitioning the system was chosen to be the C_δ_ of the target K-9. The resulting systems contained around 5500 atoms, including about 800–900 water molecules. There is a crystal water molecule in the active site of the X-ray structure, and no additional water at the active site was introduced through the solvation process.

The SCC-DFTB method [32] implemented in CHARMM [28] was used for the QM atoms and the all-hydrogen CHARMM potential function (PARAM27) [33] was used for the MM atoms. In our earlier studies, the results of the SCC-DFTB and B3LYP/6-31G** methods for the description of the methyl transfer in a small model system were compared using energy minimization-based approach [18]–[20]. This comparison allowed us to understand the performance of the semi-empirical method in the description of the bond breaking and making and derive an empirical correction for the free energy curves obtained from the potential of mean force simulations. The results indicated that the energy curves from the corrected SCC-DFTB and B3LYP/6-31G** were very close, supporting the use of this approach to make the first correction to the bond breaking and making events involving the simple and similar SN2 methyl transfer processes. The similar approach of this “first-order” correction to the free energy curves was also adopted in this work. For a more detailed discussion, see Ref.20. It should be pointed out that cares must be exercised when applying this procedure to study other methyl transfer processes involving different methyl donors and acceptors (such as the methyl transfer in protein arginine methyltransferases). In such cases, a model system containing the corresponding methyl donor and acceptor should be used instead for determining the deficiency of the SCC-DFTB method in the description of methyl transfer. The results of SCC-DFTB calculations on the methyl transfer for such model systems can be compared with the results from high-level ab initio (e.g., B3LYP/6-31G**) calculations to obtain reasonable corrections for such methyl transfer process.

The initial coordinates for the reactant complexes of the first, second and third methyl transfers were based on the crystallographic complexes (PDB codes: 2RFI, 3HNA) of GLP containing AdoHcy and short H3K9me1 or H3K9me2 peptide [15]. A methyl group was added to AdoHcy to form AdoMet. The initial coordinates for F1209Y and Y1124F mutants were generated by changing the corresponding amino acid manually. For the first methyl transfer step, methyl group in H3K9me1 was removed to generate H3K9. The initial structures for the entire stochastic boundary system were optimized using the steepest descent (SD) and adopted-basis Newton-Raphson (ABNR) methods. The systems were gradually heated from 50.0 to 283.15 K in 50 ps and equilibrated at 283.15 K for 500 ps. A 1-fs time step was used for integration of the equation of motion, and the coordinates were saved every 50 fs for analyses. After 500-ps of equilibration were performed, 1-ns QM/MM MD simulations were carried out for each of the reactant complexes of the first, second and third methyl transfers. As discussed in the previous studies [18]–[20], the S_N_2 methyl transfer from AdoMet to K9, K9me1 or K9me2 is presumably more efficient if the S-CH_3_ group of AdoMet is well aligned with the lone pair of the electrons of N_ξ_ in the reactant complex; i.e., with a small *θ* angle and relatively short C_M_-N_ξ_ distance. Therefore, we determined the distributions of *r*(C_M_-N_ξ_) and *θ* from the MD trajectories; *θ* was defined as the angle between the direction of the C_M_-S_δ_ bond (***r***_2_) and the direction of the electron lone pair on N_ξ_ (***r***_1_) (see **Figure 1**).

The umbrella sampling method [34] implemented in the CHARMM program along with the Weighted Histogram Analysis Method (WHAM) [35] was applied to determine the change of the free energy (potential of mean force) as a function of the reaction coordinate for the methyl transfer from AdoMet to H3-K9, H3-K9me1 or H3-K9me2 in the WT and mutated enzyme. The reaction coordinate was defined as a linear combination of *r*(C_M_-N_ξ_) and *r*(C_M_-S_δ_) [*R* = *r*(C_M_-S_δ_)−*r*(C_M_-N_ξ_)] (see **Figure 1**). For each methyl transfer process, twenty windows were used, and for each window 50-ps production runs were performed after 20-ps equilibration. The force constants of the harmonic biasing potentials used in the PMF simulations were from 50 to 400 kcal mol^−1^ Å^−2^. More sophisticated simulation methods [36], [37] may be used in the future to generate the free energy surfaces of methyl transfers that also depend on angular coordinates.

## Results

The active-site structure along with the average values of some structural parameters of the reactant complex for the first methyl transfer in WT GLP is shown in **Figure 2A**. As is clear from this figure, the lone pair of electrons on N_ξ_ of the target lysine is well aligned with the methyl group of AdoMet in the structure, a condition that was found to make the methyl transfer relatively easier to occur. The distribution plot from the MD simulations for the reactant complex (**Figure 2B**) also shows that there are large populations of the structures with relatively short *r*(C_M_-N_ξ_) distance and small values of the *θ* angle; the average *r*(C_M_-N_ξ_) distance is around 3.0 Å and the average *θ* angle is about 30°. **Figure 2A and 2G** (the structure near the transition state obtained from the free energy simulations along with the average values of some structural parameters) show that Tyr1124 and an active-site water molecule (W1) form stable hydrogen bonds with the ε-amino group of target lysine, and these hydrogen bonds may help to orientate the electron lone pair and achieve a good alignment with the methyl donor group of AdoMet. The active-site structure along with the average values of some structural parameters of the reactant complex for the second methyl transfer in WT GLP is given in **Figure 2C**. Similar to the case for the first methyl transfer, a good alignment between the lone pair of electrons and the methyl group from AdoMet can also be achieved in this reactant complex (also see **Figure 2D**). Examination of the structure in **Figure 2C** shows that while Tyr1124 still forms a hydrogen bond with the ε-amino group of target lysine as in the case for the first methyl transfer, the hydrogen bond with W1 disappears as a result of losing the hydrogen-bond donating capacity for the methylated lysine. It is of interest to note from the average structure near the transition state (**Figure 2H**) that W1 has dissociated from the active site during the methyl transfer. The dissociation of this water presumably produces additional space in the active site that makes the second methyl transfer easier. The active-site structure along with the average values of some structural parameters of the reactant complex for the third methyl transfer is shown in **Figure 2E**. As is demonstrated in this figure, the lone pair of electrons cannot be well aligned with the methyl group; the average *r*(C_M_-N_ξ_) distance is about 4.5 Å and the values of *θ* angle become significantly larger with a broad distribution (**Figure 2F**).

The active-site structure along with the average values of some structural parameters of the reactant complex for the first methyl transfer in the F1209Y mutant is shown in **Figure 3A**. As is the case for the wild type enzyme, the lone pair of electrons on N_ξ_ of target lysine is well aligned with the methyl group of AdoMet in the reactant complex for the first methyl transfer. **Figure 3C and 3D** show that for the second methyl transfer, the chances for the *θ* angle to reach 0–15° (i.e., near the line arrangement) become smaller in the mutant than in wild-type. Moreover, the free energy cost for generating the linear configuration is as much as 2–3 kcal/mol (only about 1 kcal/mol in wild-type, see **Figure 2D**). This energy cost may be related to the relatively higher free energy barrier for the second methyl transfer in F1209Y (see below). The structure near the transition state (**Figure 3F**) shows that unlike in the case of wild-type, W1 remains in the active site during the second methyl transfer. The failure for the dissociation of W1 from the active site is presumably due to the presence of the hydrogen bond between W1 and Y1209 (that stabilizes the water molecule), and the existence of W1 is likely to make it more difficult for further methyl addition.

The free energy profiles for methyl transfer processes in WT GLP and the F1209Y mutant are plotted in **Figure 4A and 4B**, respectively, as a function of the reaction coordinate [*R* = *r*(C_M_…S_δ_)−*r*(C_M_…N_ζ_)]. Figure 4A shows that the increase of the free energy barrier from the first or second methyl transfer to that of the third methyl transfer in WT GLP is considerably large (6–9 kcal/mol), consistent with the fact that WT GLP is a di-methylatransferase [14]. For the F1209Y mutant, there is a significant increase of the free energy barrier from the first to second methyl transfer (3.6 kcal/mol) and the chance for observing dimethyl lysine from F1209Y is expected to be much smaller compared to that for wild-type. The results are consistent with the experimental observation that F1209Y mutation altered the enzyme from a di-methyltransferase to a mono-methyltransferase [14]. These data agree with the previous conclusions that the existence of a relatively high free energy barrier could stop the further addition of methyl group and may therefore control the product specificity of PKMTs [18]–[20], at least in many cases.

The active site structures along with the average values of some structural parameters of the reactant complexes for the first, second and third methyl transfer of the Y1124F mutant are shown in **Figure 5A, 5C, 5E**, respectively. All the structures have the lone pair of electrons on N_ξ_ of target lysine well aligned with the methyl donor group of AdoMet. The distribution plots for these structures also show large population of structures with relatively short *r*(C_M_-N_ξ_) distances and small values of the *θ* angle; i.e. the average *r*(C_M_-N_ξ_) distances are around 3.0 Å and the average angles are in the range of 0–30° (**Figure 5B, 5D, 5F**). The distributions of the *r*(C_M_-N_ξ_) distance and *θ* angle for the second methyl transfer is broader compared with that observed in wild type enzyme. This could be caused by the loss of the stabilization from the hydrogen bond involving Tyr1124. Nevertheless, the free energy costs for generating the linear configuration is rather small (**Figure 5D**). The substitution of Tyr1124 by Phe may be able to lead to additional space around the ε-amino group of the target lysine and make it easier to add the third methyl group to target lysine. Indeed, the free energy profiles of all three methyl transfer steps in the Y1124F mutant in **Figure 6** show that all the three methyl transfers have similar and relatively low free energy barriers, consistent with the experimental observation that the Y1124F mutant is a tri-methyltransferase [15].

## Discussion

The results of the simulations show that the structures of the reactant complexes for the first and second methyl transfers in WT (**Figures 2A, 2C**), the first methyl transfer in F1209Y (**Figure 3A**), and the first, second and third methyl transfers in Y1124F (**Figures 5A, 5C, 5E**) are rather similar to the corresponding structures near transition state (TS). The distribution plots (**Figure 2B, 2D** **,** **3B** **,** **5B, 5D, 5F**) of these structures show large population of structures with relatively short *r*(C_M_-N_ξ_) distances and small values of the *θ* angle, which are close to the distribution plots (**Figure S1**) of the TS structures. This is in contrast to the case of the third methyl transfer in wild-type and the second methyl transfer in F1209Y mutant where the reactant structures (**Figure 2E** **,** **3C**) are significantly distorted from the corresponding TS structures (**Figure 2I** **,** **3F**). The distribution plots (**Figure 2F** **,** **3D**) of these reaction complexes show large population of structures with either relatively long *r*(C_M_-N_ξ_) distances or large values of the *θ* angle, which are considerably different from the corresponding distribution plot (**Figure S1**) of the TS structures. Thus, the results are consistent with the previous suggestion [18]–[20] that one of the reasons for the existence of the relatively low free energy barrier is likely owed to the fact that a part of TS stabilization is already reflected on the reactant state through the generation of the TS-like conformation. Thus, a good alignment of the electron lone pair on N_ξ_ of target lysine with the methyl group of AdoMet is likely to lead to a relatively efficient methyl transfer, while a poor alignment could make the methyl transfer more difficult.

Two free energy triplets, (0, Δ_2-1W_, Δ_3-1W_) and (Δ_M-W_, Δ_2-1M_, Δ_3-1M_) have been proposed [18]–[20] to describe the energetics of the methyl transfers for PKMTs and their mutants, respectively, and to determine their product specificity. Here the free energy barrier of the first methyl transfer step in WT enzyme is taken as the zero. For WT enzyme, the second (Δ_2-1W_) and third (Δ_3-1W_) parameters are the differences in the free energy barriers between the second and first and between the third and first methyl transfers, respectively. These two parameters therefore provide information about the relative efficiencies of the second and third methyl transfers compared to the first methyl transfer. For instance, if Δ_2-1W_ is significant (e.g., in the case of SET8 [19]), the enzyme may stop the methylation process leading to a mono-methylase. For the mutated enzyme, the first parameter (Δ_M-W_) is the difference in the free energy barriers for the first methyl transfer in the WT and mutant. The second (Δ_2-1M_) and third (Δ_3-1M_) parameters are the differences in the free-energy barriers between the second and first and between the third and first methyl transfer, respectively (e.g., defined similar to the cases for WT). Based on such definitions, the energy triplets for WT, F1209Y and Y1124F mutants of GLP are (0, 2.4, 8.7), (0.4, 4.0, x) and (0.5, 2.5, −0.1), respectively (**Figure 4** **and** **6**). The significant increase of the free energy barrier from the first or second methyl transfer to that of the third methyl transfer in WT GLP is consistent with the observation that the enzyme is a di-methylase based on the simulations. For the F1209Y mutant, the free energy barrier of the second methyl transfer is around 3.6 kcal/mol higher than the first methyl transfer, and the mutant is more likely to be mono-methylase. It is of interest to note that the free energy barriers of all three methyl transfers in the Y1124F mutant are similar, suggesting that the replacement of Y1124 by F changes the enzyme from a di-methylase into a tri-methylase. All these results are consistent with previous experimental observations [14], [15].

The structural information generated from the simulations shows that the dissociation of W1 might occur during the second methyl transfer in both WT GLP and the Y1124F mutant (**Figure 2H** **,** **5H**). For the second methyl transfer in the F1209Y mutant, however, W1 cannot dissociate from the active site, presumably, due to its formation of the hydrogen bond with Tyr1209. Consequently, the free energy barrier increases significantly for the addition of the second methyl group, leading to a mono-methylase. These results are consistent with the earlier suggestion that the dissociation of the active site water molecule is important for further addition of methyl group to target lysine [13], [17]–[19]. For the Y1124F mutant, additional space in the active site may be generated by the removal of the hydroxyl group on Y1124, making it easier for the addition of the third methyl group.

### Conclusion

In this study, the QM/MM MD and free energy simulations have been performed on different methyl transfer steps from AdoMet to target lysine/methyl-lysine in WT GLP as well as its F1209Y and Y1124F mutants. The reactant complexes for different methyl transfers generated from QM/MM MD simulations support the suggestion that the ability of the reactant complex to form the TS-like reactive configuration (in which the electron lone pair on N_ξ_ of target lysine is well aligned with the methyl group of AdoMet) is important for the methyl addition. The free energy barriers of methyl transfers were also calculated. The results show that the relative free energy barriers for the three methyl transfers may determine the product specificity for WT GLP and its mutants. The role of the active site water molecule in controlling product specificity was also discussed.

## Supporting Information

Figure S1 **The two-dimensional plots of** ***r*** **(C_M_…N_ζ_) and** ***θ*** **distributions of the transition state complexes.** A: plots for WT GLP, left: first methyl transfer; middle: second methyl transfer; right: third methyl transfer. B: plots for F1209Y mutant, left: first methyl transfer; right: second methyl transfer. C: plots for Y1124F mutant, left: first methyl transfer; middle: second methyl transfer; right: third methyl transfer. All distribution plots are based on the 50-ps production runs of the transition state complexes.
(TIFF)
