# Supplementary figures and images for: QM/MM MD and Free Energy Simulations of G9a-Like Protein (GLP) and Its Mutants: Understanding the Factors that Determine the Product Specificity
Source: PLoS One. 2012 May 18;7(5):e37674. doi: 10.1371/journal.pone.0037674 (PMC3356298; doi:10.1371/journal.pone.0037674)

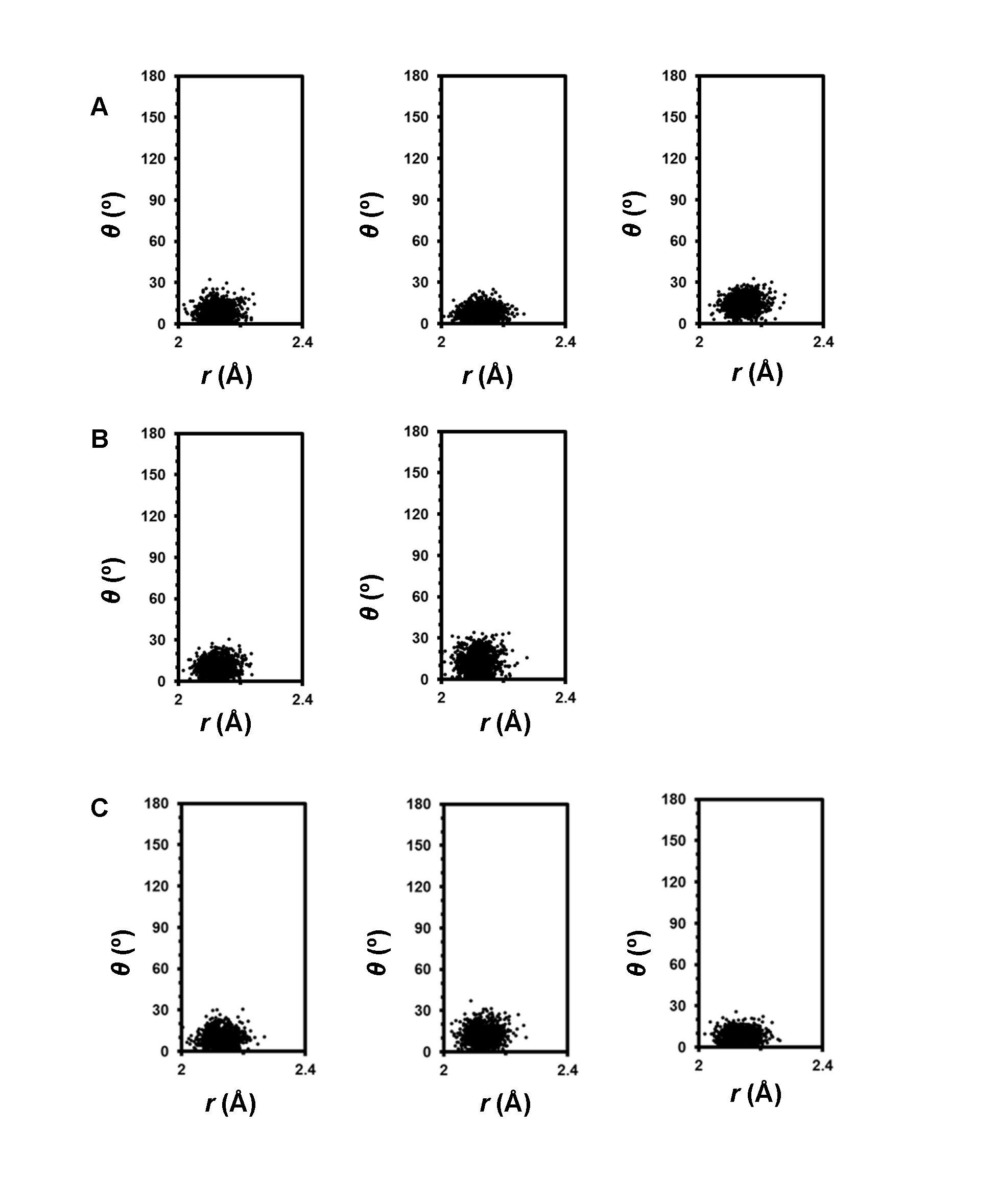

Supplement: Figure S1 — The two-dimensional plots of r (CM…Nζ) and θ distributions of the transition state complexes. A: plots for WT GLP, left: first methyl transfer; middle: second methyl transfer; right: third methyl transfer. B: plots for F1209Y mutant, left: first methyl transfer; right: second methyl transfer. C: plots for Y1124F mutant, left: first methyl transfer; middle: second methyl transfer; right: third methyl transfer. All distribution plots are based on the 50-ps production runs of the transition state complexes. (TIFF) [file pone.0037674.s001.tif]
